# Post-Kala-Azar Dermal Leishmaniasis

**DOI:** 10.4269/ajtmh.24-0018

**Published:** 2024-07-23

**Authors:** Apoorva Sharma, Anuradha Bishnoi, Davinder Parsad

**Affiliations:** Department of Dermatology, Venereology and Leprology, Postgraduate Institute of Medical Education and Research, Chandigarh, India

A man in his 20 s presented with multiple, asymptomatic, variably sized, well-defined, hypopigmented patches on his neck and trunk for the past 8 years (Figure 1). He also had multiple discrete, nontender, firm, skin-colored to slightly erythematous papulonodular lesions on the central part of face, ears, limbs, and back, varying in size from few millimeters to some reaching 1–2 cm for the past 6 years. There was a grouping of papulonodules over the nose and mouth area of the face (Figure 2). There was no loss of appendages or feeding nerve localized to the hypopigmented plaques. There was no hypoesthesia within the hypopigmented patches. Nerves were not enlarged. There was no other significant past history.

**Figure 1.**
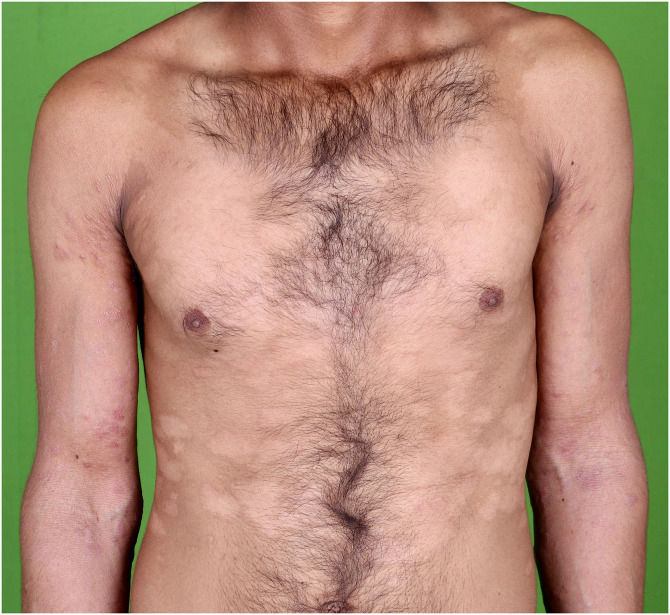
Multiple, well-defined, non-scaly, normoesthetic, hypopigmented patches ranging in size from 3 × 5 cm to 10 × 15 cm over the trunk, neck, and back.

**Figure 2.**
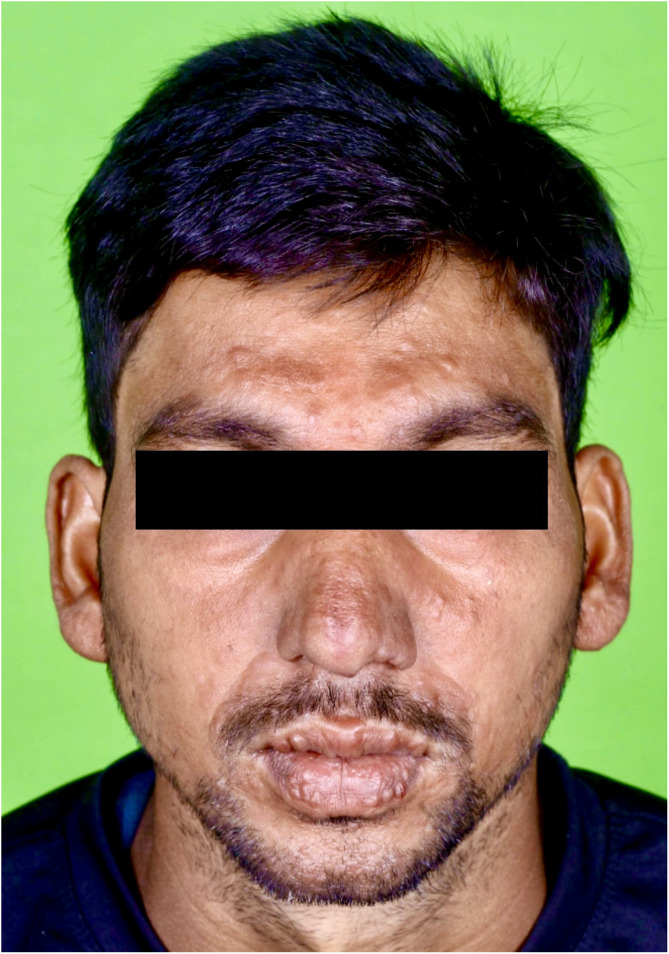
Multiple discrete, skin-colored to slightly erythematous papulonodular lesions on the central part of the face, ears, upper limbs, and back, ∼1 to 2 cm. These lesions covered the nose and mouth area of the face, which may be an important clinical clue for the diagnosis of post-kala-azar dermal leishmaniasis.

Laboratory investigations showed positive IgG serology for rk39 antibody test (rk39 antibody detection test kits by K.N.D. Research Solution, SAS Nagar, Mohali, India). Skin biopsies showed presence of basal cell vacuolisation. The dermis showed mixed inflammatory infiltrates comprising lymphocytes, histiocytes, plasma cells, and neutrophils. There was presence of well-formed epithelioid cell granulomas with scattered multinucleated giant cells. The histiocytes showed intracytoplasmic amastigote forms of leishmania (Figure 3A and B), Giemsa stain showed presence of Leishman–Donovan bodies (Figure 3C). Polymerase chain reaction from skin biopsy for leishmania was positive (positive control used was DNA from cultured promastigotes, negative control was nucleus free water). A diagnosis of post-kala-azar dermal leishmaniasis (PKDL) was established based on clinical, laboratory, and histopathological findings. The patient was treated with intravenous liposomal amphotericin B 2.5 mg/kg/day for 20 days, and there was significant reduction in all skin lesions at end of therapy (Supplemental Figure 1).

**Figure 3.**
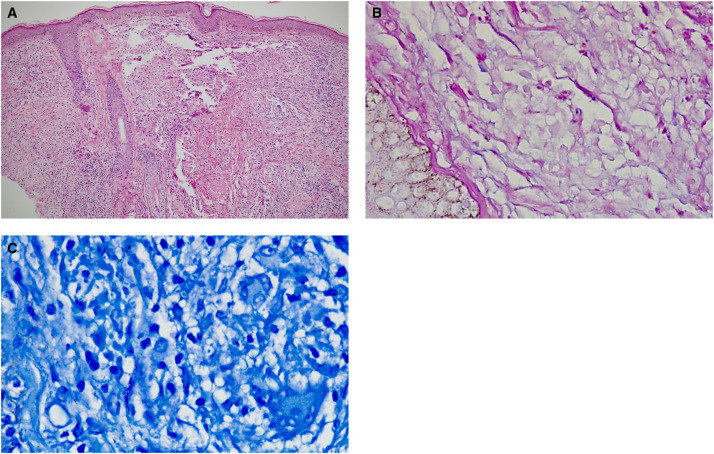
(**A** and **B**) Skin biopsies showed presence of Grenz zone and band-like infiltrate in papillary dermis. Dermis showed mixed inflammatory infiltrates comprising of lymphocytes, histiocytes, plasma cells, and neutrophils. There was presence of well-formed epithelioid cell granulomas with scattered multinucleated giant cells. The histiocytes showed intractoplasmic amastigote forms of leishmania (hematoxylin and eosin stain, 40× and 100×) (**C**) Giemsa stain showed presence of Leishmanin–Donovan bodies (100×).

This is a case of PKDL without a history of visceral leishmaniasis (VL), which is particularly difficult to diagnose. PKDL without recognized VL is a form leishmaniasis that has garnered increasing attention in India as well as other parts of the world in recent years.^1^^,^^2^ Such cases are due to asymptomatic or subclinical *Leishmania donovani* infection.^3^ However, PKDL occurs after VL only in a subset of people, and factors implicated in its cause include exposure to antimonial therapy and ultraviolet radiation (UVR); UVR acts as a potent immunosuppressant, induces production of IL-10, and alters the functioning of the antigen presenting cells. Morphologies of PKDL include hypopigmented, papulonodular, and polymorphic types, the latter of which includes multiple lesion morphologies such as papules, plaques, nodules, and hypopigmented patches. Diffuse cutaneous leishmaniasis (DCL), caused by *Leishmania amazonensis*, is a rare form of cutaneous leishmaniasis where parasites grow uncontrolled in lesions across large skin areas. Leishmania-specific immunity is higher in cases of PKDL than DCL, and rates of parasite positivity are lower in PKDL than DCL. Lepromatous leprosy remains a close differential diagnosis of PKDL in VL-endemic regions. Subtle features on clinical examination such as involvement of the nose and mouth area, lack of sensory loss, and involvement of warm and cool areas of the body (like the spine) in PKDL helps in differentiating it from various forms of leprosy, which are typified by hypopigmented, anesthetic skin lesions. rk39 serology is often used for complementing diagnosis in PKDL; however, an important limitation of this test is that it cannot distinguish between current and past infection.

PKDL with or without antecedent VL is complex in terms of epidemiology, diagnosis, and management. The emergence of PKDL without history of VL in India indicates evolving changes in our understanding of the disease, enabled by molecular diagnosis. A high level of clinical suspicion combined with enhanced diagnostic tools is essential for developing targeted control strategies for PKDL and VL control in endemic areas.

## Supplemental Materials

10.4269/ajtmh.24-0018 Supplemental Materials
