# Supplementary figures and images for: Post-Kala-Azar Dermal Leishmaniasis
Source: Am J Trop Med Hyg. 2024 Jul 23;111(4):710–2. doi: 10.4269/ajtmh.24-0018 (PMC11448539; doi:10.4269/ajtmh.24-0018)

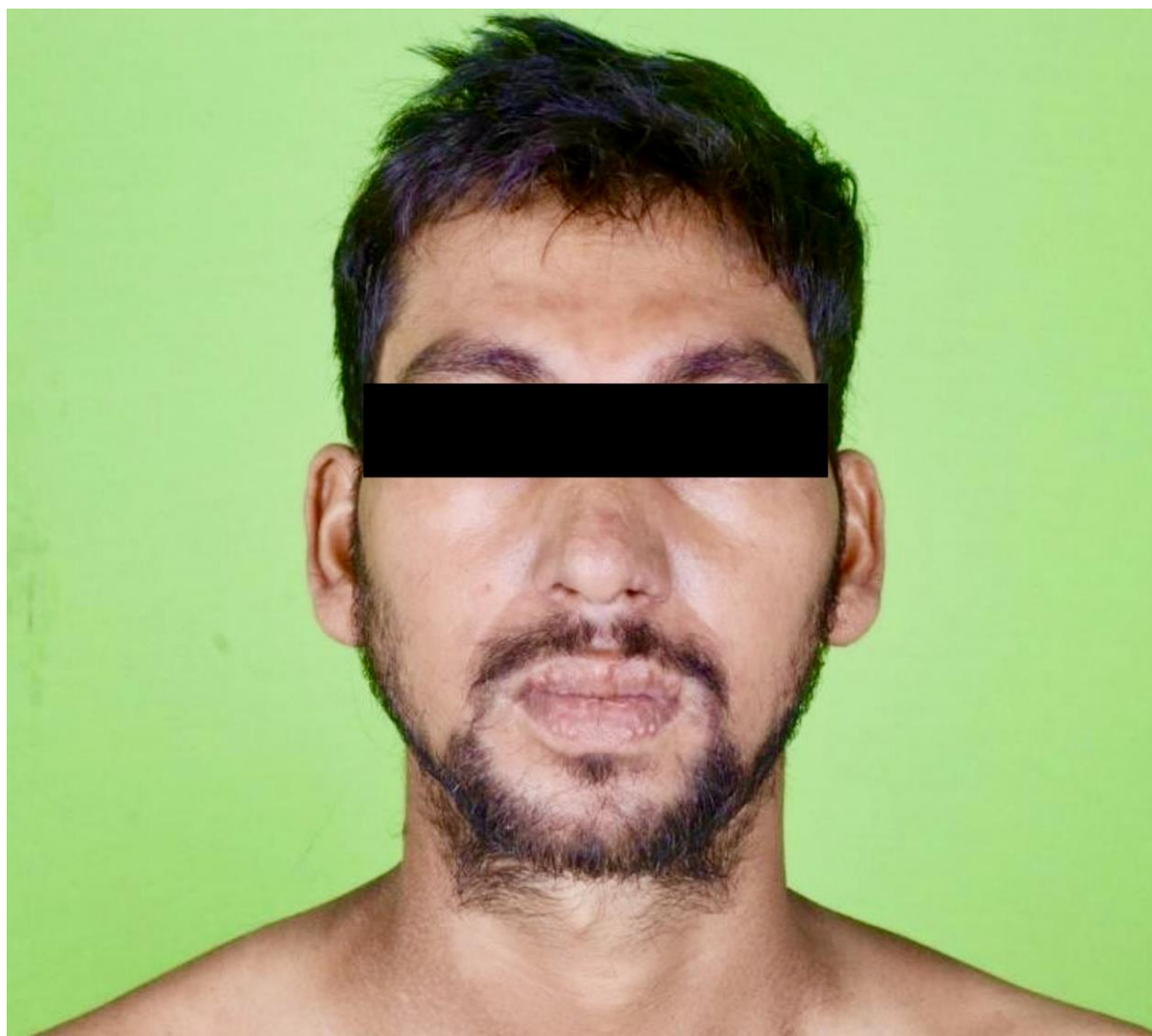

Supplement: Supplemental Materials [file tpmd240018.SD1.pdf]
